# Case Report: Incidental late-onset Pompe disease diagnosis in a man with no clinical and instrumental evidence of neuromuscular dysfunction

**DOI:** 10.3389/fgene.2025.1574381

**Published:** 2025-06-23

**Authors:** Monica Sciacco, Sabrina Lucchiari, Letizia Bertolasi, Giacomo Pietro Comi, Stefania Corti, Dario Ronchi

**Affiliations:** ^1^ Neuromuscular and Rare Disease Unit, Fondazione IRCCS Ca’ Granda Ospedale Maggiore Policlinico, Milan, Italy; ^2^ Department of Pathophysiology and Transplantation, Dino Ferrari Center, University of Milan, Milan, Italy; ^3^ Neurology Unit, IRCCS Fondazione Ca' Granda Ospedale Maggiore Policlinico, Milan, Italy

**Keywords:** Pompe disease, GAA, muscle glycogenosis, IVS1-32-13T>G, acid maltase deficiency

## Abstract

Glycogen storage disease II or Pompe disease (PD), is a rare autosomal recessive disorder due to biallelic pathogenic variants in *GAA*, resulting in the enzymatic deficiency of alpha-1,4-glucosidase. Two clinical forms are recognized, namely, early onset (EOPD) and late-onset (LOPD). We present the case of an asymptomatic 33-year-old man who underwent a genetic screening for autosomal recessive disorders (parental prenatal counselling) and was found to carry the homozygous pathogenic *GAA* substitution NM_000152.5(GAA):c.-32-13T>G (IVS1). Neurological examination, serum CK levels, electromyography, muscle MRI, respiratory and cardiac screening were reported normal. We investigated the effects of the variant at transcript and protein levels in available tissues from the proband and his parents. The IVS1-32-13T>G variant (dbSNP: rs386834236, Clin Var ID: 4,027) occurs in 90% of Caucasian LOPD patients and is associated with a broad range of symptom onset. About 50 subjects have been reported harboring this variant in homozygosis and most of them are asymptomatic, although a subset develops symptoms with time. Residual levels of alpha-1,4-glucosidase activity and protein content do not seem to reflect clinical severity in homozygous IVS1 LOPD patients.

## 1 Introduction

Glycogen storage disease II (GSDII, OMIM 232300), or Pompe disease, is a rare autosomal recessive disorder caused by the enzymatic deficiency of the alpha-1,4-glucosidase (GAA or acid maltase) which is deputed to the glycogen degradation within lysosomes (Stevens et al., 2022).

Two main clinical forms are recognized, based on the age of symptom onset. Early-onset Pompe disease (EOPD) manifests at birth or before 12 months of age with severe muscular hypotonia and hypertrophic cardiomyopathy. Late-onset Pompe disease (LOPD) is more unpredictable, initial symptoms occurring anytime in childhood, juvenile or adult years. Also, LOPD clinical manifestations are highly heterogenous with variable degrees of severity and progression rate. Cardiac involvement is generally absent, respiratory muscles being almost always affected (Barba-Romero et al., 2012).

GSDII is caused by biallelic molecular defects in the *GAA* gene (OMIM *606800), located on chromosome 17. More than 500 variants, detected in the whole *GAA* coding sequence, were reported as pathogenic (Peruzzo et al., 2019).

Clinical heterogeneity only partially correlates with residual enzymatic activity, or the type of molecular defects found in patients, which contributes to diagnostic delay (mean age at diagnosis: 27–41 years in adult LOPD patients) (Kishnani et al., 2013).

Approximately 90% of Caucasian LOPD patients have been reported to carry the splicing mutation NM_000152.5(GAA):c.-32-13T>G (IVS1) (Boerkoel et al., 1995), generally found *in trans* with different pathological alleles (at least 67 so far described). Mean age at symptom onset for IVS1 carriers is 30 years (range between 1 and 71 years) (Niño et al., 2021a). IVS1 (dbSNP: rs386834236, Clin Var ID: 4,027) is referred to as a leaky splicing variant since it promotes the inefficient inclusion of Exon 2 in the mature *GAA* transcript, favoring the increase of alternative splicing products (Bergsma et al., 2019), preserving the production of some transcripts that are correctly spliced.

So far, about 50 patients have been described harboring the IVS1 defect on both alleles (Supplementary Table 1) and most of them have been recently identified by the implementation of GSDII newborn screening program in several countries (Sawada et al., 2020; Rairikar et al., 2017). Subjects homozygous for the IVS1 variant might develop clinical symptoms with time while others remain asymptomatic (Musumeci et al., 2015). Similarly, residual GAA activity changes significantly in different tissues from the same patient and does not reliably reflect the severity of the disease among LOPD patients, confirming seminal observation collected in the pre-genetic era (Mehler and DiMauro, 1977).

Few reports have addressed the correlation between the IVS1 homozygous genotype, the rate of Exon 2 inclusion and the residual levels of GAA protein and enzymatic activity (Bergsma et al., 2019). This information might be helpful to implement genetic counselling of this (likely underestimated) defect.

Beside newborn screening programs, the detection of the variant is also facilitated by NGS genetic testing performed for prenatal counselling purpose in unaffected probands.

Here we describe one of these “fortuitous” cases and we investigate the effects of the variant at transcript and protein level in available tissues from both the proband and his parents.

## 2 Methods

The study was approved by the institutional review board of the Fondazione IRCCS Ca’ Granda Ospedale Maggiore Policlinico. The subjects involved provided written informed consent for all aspects of the study.

*GAA* molecular screening was performed by Sanger sequencing on an ABI prism 3,130 instrument. Total RNA was extracted from patient’s and control fibroblasts by using the Relia Prep RNA Miniprep System (Promega). The same method was used for RNA extraction in lymphocytes isolated from the proband, his parents and healthy controls. RNA samples were retrotranscribed by using the Maxima Reverse RT Master Mix (Life Technologies).

RT-PCR analysis of the transcript region encompassing Exon 2 was performed by using the following primers: FOR: 5′-AGG​TTC​TCC​TCG​TCC​GCC​CGT​T; RC: 5′-TCC​AAG​GGC​ACC​TCG​TAG​C. RT-PCR amplicons were electrophoresed on 2.5% agarose gel and on an Agilent Tape Station 4,200 instrument (D1000 Screen tape and reagent). Quantitative RT-PCR experiments were performed by using Taqman-based detection on an ABI 7500 Real Time PCR systems by using a relative quantification approach. The following probes were used: Hs00164635_m1 (*GAA*, exon junction 1-2); Hs01089838_m1 (*GAA*, exon junction 4-5); Hs99999903_m1 (*ACTB,* encoding the housekeeping beta actin). Each sample was run in triplicate.

Acid maltase activity was assessed by fluorometric method using the substrate 4-methylumbelliferyl-α-d-glucoside in lymphocytes and fibroblasts. Protein levels of GAA were assessed by SDS-PAGE Western blot analysis on a 4%–12% gradient polyacrylamide gel. The signals of the antibodies directed against GAA (Abcam, ab137068, 1:1,200) and GAPDH (Santa Cruz biotechnology, sc-20357, 1:7,000), for normalization purpose, were acquired by using the Licor Odyssey Platform and quantified by densitometry (Licor Image Studio).

## 3 Case description and results

**FIGURE 1:**
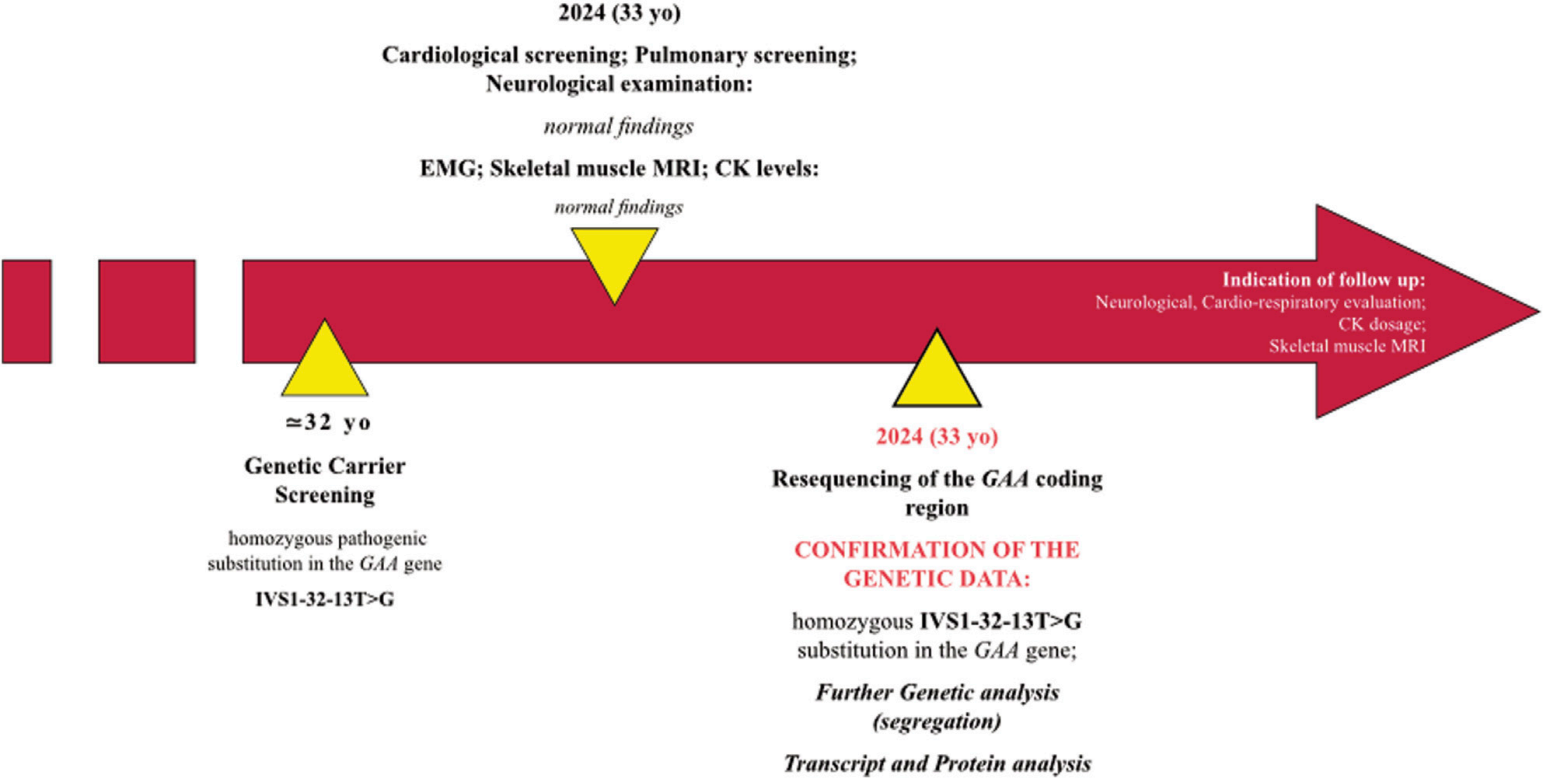
Timeline with relevant data of the case described.

The proband is a 33-year-old man with no previous medical history (Figure 1). He does not smoke and occasionally drinks wine with meals. Along with his wife (4 months pregnant), he underwent prenatal genetic testing for AR disorders (private GENOMICA Institute, Rome) and was found to carry a homozygous pathogenic IVS1-32-13T>G mutation on the *GAA* gene which is consistent with a diagnosis of LOPD. The patient has no neuromuscular symptoms (no weakness, myalgia or cramps) nor does he report limitations in his daily life, including practice of regular physical activity. He refers occasional four limb paresthesia. His mother reports that his son had occasionally complained of “leg pain” as a child (at the age of 2–3 years). Cardiological screening, including EKG, EchoKG and subsequent cardiological evaluation, respiratory assessment based on spirometry and blood gas analysis, as well as serum CK levels are all normal (CK values fluctuating between 80 and 160 U/L). His parents are non-consanguineous but come from two towns in the province of Frosinone, 30 km away from each other. The patient is an only child. His wife has no mutations in the *GAA* gene, the fetus, heterozygous for the mutation, is thus a compelled disease carrier.

When he came to our observation, we performed neurological examination which was completely normal. Also, no abnormalities were found at both EMG examination, skeletal muscle MRI and respiratory full screening.

Given the presence of a clinical and instrumental benign condition, we did not give any indication to start an enzyme replacement therapy, and we suggested adhering to the follow-up schedule proposed for LOPD-diagnosed newborns (Gragnaniello et al., 2022). In this regard, we recommended patient and serum CK levels evaluation every 6–8 months and yearly monitoring of the cardio-respiratory status. If the condition remains stable, skeletal muscle MRI should be performed every 2 years.

**FIGURE 2:**
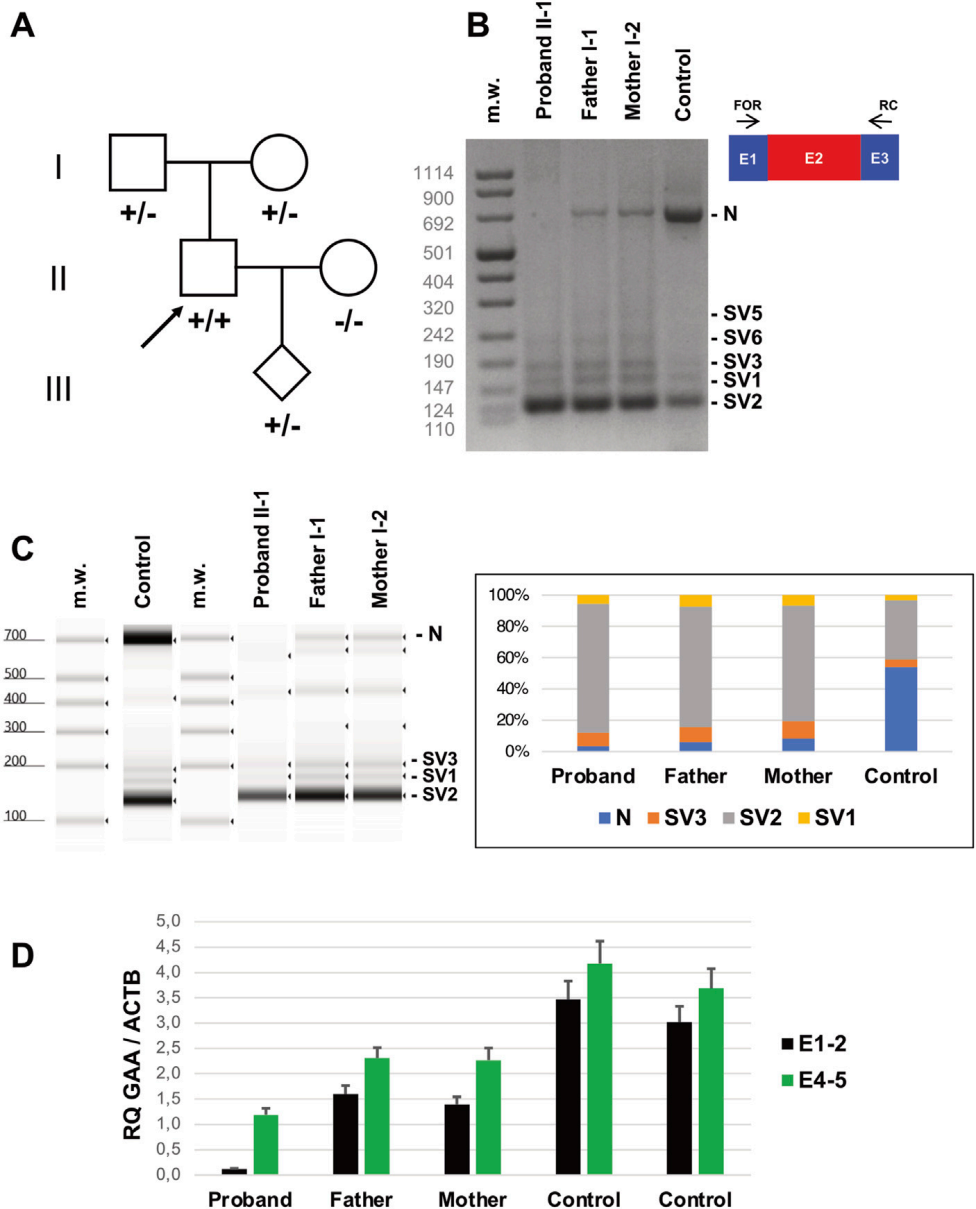
Genetic studies **(A)** Pedigree of the family investigated. Subjects harbouring IVS1-32-13T>G in homozygosis (+/+) or heterozygosis (+/−) are shown. The arrow indicates the Proband. **(B)** Reverse transcription PCR (RT-PCR) encompassing *GAA* transcript Exons 1 (E1) and 3 (E3) in lymphocytes of the subjects indicated. Amplicons corresponding to normally spliced molecules including Exon 2 (“E2” = Normal transcript “N”) and altered transcripts (Splice Variants “SV1-3”) are indicated. **(C)** Tape Station analysis of RT-PCR amplicons and their relative quantification. **(D)** Quantitative RT-PCR analysis of *GAA* transcripts in lymphocytes of the subjects indicated as evaluated by Taqman probes targeting Exons 1-2 and 4-5 junctions.

We resequenced the *GAA* coding region in the proband and confirmed the presence of the “common” pathogenic substitution IVS1-32-13T>G in homozygosis. The variant was found in heterozygosis in proband’s parents (Figure 2A). No additional rare variants were detected in the *GAA* region and the proband was negative for the synonymous variant c.510C>T which had been demonstrated to act as a genetic modifier of the IVS1-32-13T>G mutation (Bergsma et al., 2019).

Biochemical analysis in lymphocytes disclosed the 93% reduction of alpha-1,4-glucosidase activity in the proband (1.07 nmol/mg, normal range: 15–50 nmol/mg) and 30% of reduction in his parents (11.00 nmol/mg and 10.00 nmol/mg in his father and mother, respectively).

We checked the levels of transcripts including Exon 2 in lymphocytes collected from the proband and his parents (Figure 2B). Qualitative RT-PCR experiments showed a severe reduction of GAA transcript including Exon 2 in the proband’s blood lymphocytes, compared to controls. Multiple amplicons corresponding to alternative splicing products, including the previously identified transcripts SV1, SV2 and SV3, were evident in proband’s cells but also detectable in control samples, as previously observed (Wal et al., 2017a). Proband’s parents displayed intermediate levels of Exon2-including transcripts in the range between the proband and the control samples, as detected by quantitative analysis of electrophoresed fragments (Figure 2C).

**FIGURE 3:**
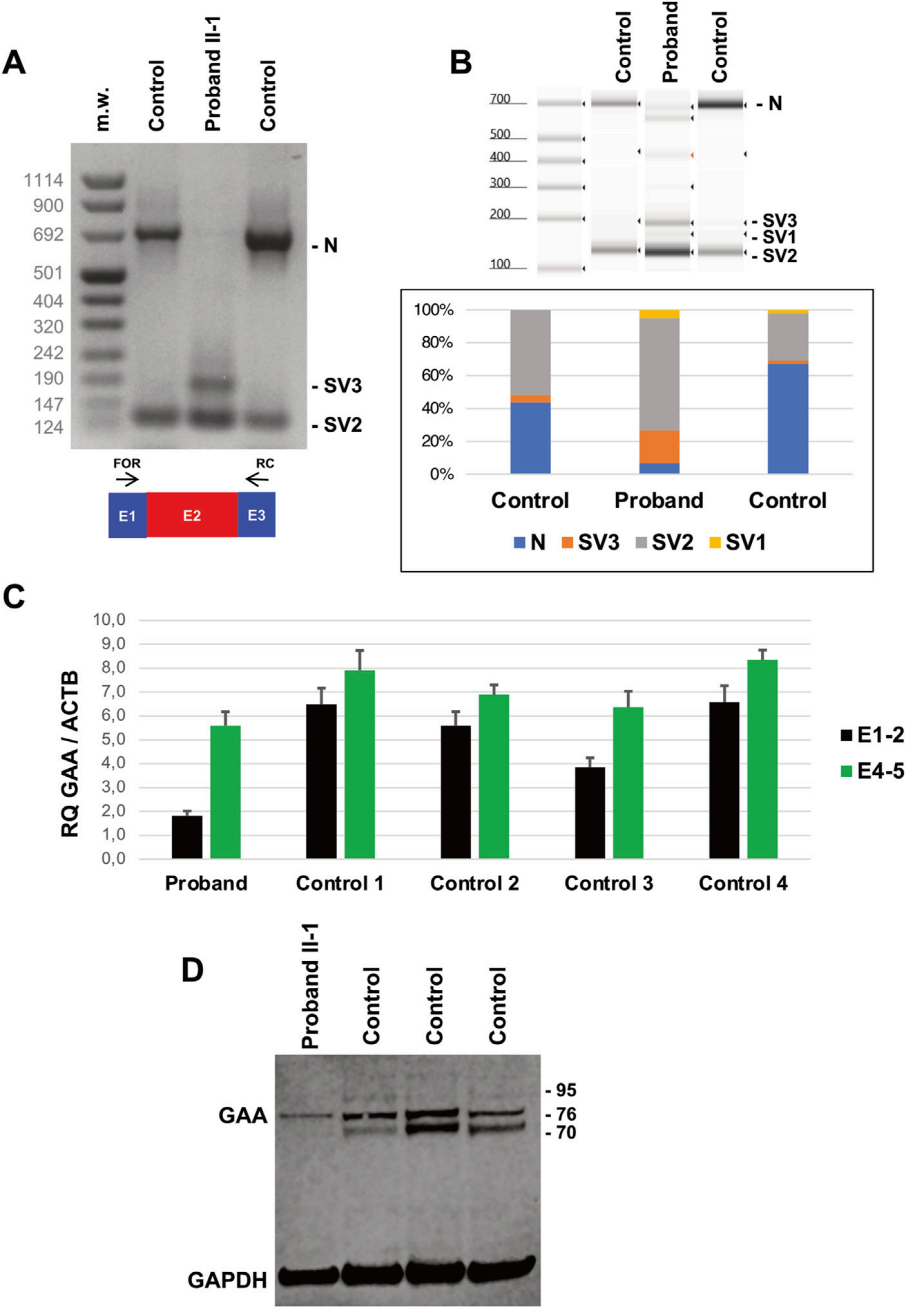
Transcript and Protein analysis of Patients’ fibroblasts. **(A)** Reverse transcription PCR (RT-PCR) encompassing *GAA* transcript Exons 1 (E1) and 3 (E3) in Proband’s and control fibroblasts. Amplicons corresponding to normally spliced molecules including Exon 2, (“E2” = Normal transcript “N”) and altered transcripts (Splice Variants “SV1-3”) are indicated. **(B)** Tape Station analysis of RT-PCR amplicons and their relative quantification. **(C)** Quantitative RT-PCR analysis of *GAA* transcripts in Proband’s and control fibroblasts as evaluated by Taqman probes targeting Exons 1-2 and 4-5 junctions. **(D)** Western blot analysis of GAA protein levels in Proband’s and control fibroblasts. Mature forms of GAA enzyme correspond to bands at 76 and 70 kDa. GAPDH levels are used for normalization purpose.

Quantitative RT-PCR evaluation of *GAA* transcript in lymphocytes showed a severe loss (<5% of controls) of molecules including Exon 2 in the Proband (Figure 2D). Reduction was less evident in Proband’s parents (40%–50% compared to controls). Overall, quantitative levels of *GAA* transcript (assessed by targeting Exons 4-5 junction) were also reduced in the Proband’s compared to controls. These investigations were replicated in skin fibroblasts collected from the Proband and healthy subjects. The inclusion of Exon 2 was severely affected in mutated cells compared to controls, as observed by RT-PCR in qualitative (Figures 3A,B) and quantitative (Figure 3C) experiments. Protein studies revealed a significant reduction of the bands at 76 and 70 kDa representing the mature (active) forms of alpha-1,4-glucosidase (Figure 3D).

Biochemical analysis showed reduced residual acid maltase activity in the Proband’s fibroblasts 10.46 pmol/min/mg compared to controls (370.76 ± 128.91 pmol/min/mg).

## 4 Discussion

Here we discuss the molecular and biochemical findings in a trio presenting the *GAA* IVS1-32-13T>G allele. The Proband is an asymptomatic 33-year-old man presenting the IVS1 variant in homozygosis. His parents are unaffected carriers.

About 50 subjects have been so far described harboring the IVS1 variant in homozygosis (Supplementary Table 1). Thirty-one (including our proband) are reported asymptomatic while 19 patients display a mean age at onset of clinical symptoms of 39.63 ± 14.89 years (minimum 12 years, maximum 64 years). It should be noted that half of the homozygous IVS1 subjects (n = 23, 46%) were identified by newborn screening programs in the last 5 years and none of them has been reported symptomatic so far. Among the clinically affected subjects, proximal muscle weakness was reported in 89.5% of patients. None of them displayed the EOPD phenotype. Cardiological abnormalities were not reported while progressive respiratory involvement was frequent (half of the symptomatic subjects) and insidious: a patient developed moderate respiratory insufficiency at the age of 49 years, only 2 years after the onset of motor symptoms (Müller-Felber et al., 2007).

Although IVS1 is commonly regarded as a “mild” mutation, heterogenous clinical presentations can be observed in homozygous patients ranging from isolated myalgia and cramps in a 54-year-old man (Musumeci et al., 2015; Sharma et al., 2005) to moderate or severe skeletal muscle weakness usually observed since the fourth decade or earlier (Müller-Felber et al., 2007; Gal et al., 2021; Sc et al., 2007). Indeed, Musumeci et al. described six adult LOPD patients presenting myalgia, hyperCKemia, and/or exercise induced fatigue, with symptom onset between the second and the sixth decade (Musumeci et al., 2015). In another report, four patients were identified by newborn screening and classified as essentially asymptomatic when examined at 3 months of age. However, subtle motor involvement was spotted only after a vigilant disease-specific approach to evaluation. Notably, one of the patients also showed feeding and swallowing difficulties, two features usually observed in EOPD patients (Rairikar et al., 2017).

Increased serum creatine kinase levels were observed in half of the subjects in which they were measured. EMG was normal in asymptomatic or paucisymptomatic subjects (Musumeci et al., 2015; Echaniz-Laguna et al., 2015) whereas a myopathic pattern was displayed in clinically affected patients (Müller-Felber et al., 2007; Sharma et al., 2005).

We were unable to find a clear correlation between acid maltase residual activity and clinical phenotype in IVS1 homozygous subjects. Indeed, available data collected in different study groups are highly heterogenous in terms of examined tissue (lymphocytes, fibroblasts, skeletal muscle) and enzyme assay methods which prevents us from making any reliable correlations.

In addition, most of the (so far disclosed) asymptomatic subjects have been detected by perinatal DBS activity which is a screening (non-diagnostic) test. GAA activity levels ranged from 12% to 40% of controls even in muscle of IVS1 homozygous subjects. Unexpectedly, activity was found very low (13% of controls) (Musumeci et al., 2015) or even absent (Echaniz-Laguna et al., 2015) in muscles from clinically asymptomatic subjects presenting hyperckemia. These aspects highlight the issue of the lack of consensus guidelines for biochemical testing in Pompe disease, although multi-year experience from reference laboratories (Niño et al., 2021b) or recommendations for Pompe diagnosis by international network of distinguished centers for Metabolic disorders (Parenti et al., 2024) are expected to overcome this limitation.

The IVS1 variant produces the partial skipping of exon 2 during *GAA* pre‐mRNA splicing, as described previously (Wal et al., 2017a; Dardis et al., 2013; Wal et al., 2017b). The heterogeneity of the clinical manifestations of IVS1 genotype clearly reflects the existence of additional contributors able to modify the behavior of the variant, as molecular changes acting in cis with the mutated allele. An example is given by the c.510C>T variant which was demonstrated to affect the levels of normally spliced transcripts in IVS1 patients, further reducing residual GAA activity (Bergsma et al., 2019). In our family, this detrimental change was not detected.

In the investigated tissues, *GAA* overall transcript levels were found reduced proportionally to the number of mutated alleles. This is also confirmed by residual GAA activity levels that are lower than in controls. The residual enzymatic activity originating from IVS1 alleles has been estimated to be of 10%–15% per allele, resulting from the leakage of normally spliced mRNA and sustaining the patient to adult life. (Boerkoel et al., 1995; Hule et al., 1994). Although the assessment of GAA activity in peripheral cells is less reliable compared to muscle analysis (Schoser et al., 2024), the levels of acid maltase activity detected in our proband seem very low compared to previously reported patients. It should be noted that, in our proband, the levels of transcript undergoing normal splicing are like those observed in another subject carrying the homozygous IVS1 variant without the c.510C>T modifier and presenting an overlapping residual enzymatic activity in fibroblasts (Patient 20) (Bergsma et al., 2019). This patient had developed mild myopathic involvement (cramps and myalgia) at 49 years of age, anticipating a possible clinical involvement in our Proband, whose medical assessment is currently unremarkable.

The c.‐32‐13T>G is the most reported variant in LOPD patients from Europe, North and Latin America while is less common In Asia and Middle East (Reuser et al., 2019). About 90% of the Caucasian LOPD patients display at least one pathogenic allele harboring the IVS1 variant (Niño et al., 2019). Nevertheless, the incidence of the IVS1 variant is likely underestimated. Its MAF in the Caucasian population is estimated to be as high as 0.8% (Niño et al., 2019) meaning that many subjects presenting the variant in homozygosis are unaffected or only display a mild or subclinical involvement that could be underdiagnosed (Rairikar et al., 2017). Worldwide population allelic frequency in GnomAD for IVS1 variant is 0.005255 with a gradient from East Asian (MAF: 0.0001129) to European (non-Finnish) population (MAF: 0.006355), occurrence being equal in males and females. GnomAD reports 24 IVS1 homozygous subjects for whom clinical information is not available.

The increasing use of broad genetic analysis in a diagnostic setting (Lévesque et al., 2016) will likely expand the number of subjects harboring this pathogenic allele, increasing our knowledge about the clinical consequence of the variant when found in homozygosis or heterozygosis in compound with a different molecular defect. Overall, findings collected on IVS1 homozygous patients suggest that this genotype is not associated with a severe course of the disease. However, given the existence of a therapeutic option, an in-depth follow up of these patients is mandatory. ERT starting is likely not necessary for adult patients while it should be evaluated on symptomatic pediatric cases. The decision on when to start ERT in pre-symptomatic LOPD patients is a treatment dilemma in the balance between the expected relevant benefits in terms of muscle preservation and medical and economical aspects, namely, risk of side effects, long-term immunogenicity and costs for health systems.

In the case we presented, the clear evidence of the detrimental effects of the IVS1 variant on *GAA* transcript organization as well as alpha-1,4-glucosidase stability and activity does not match the absence of symptoms, the patient thus joining the small group of homozygous IVS1 carriers reaching adulthood without developing symptoms. Absence of symptoms argues against starting ERT administration in this proband, but makes personalized clinical surveillance over time mandatory. In this regard, monitoring of asymptomatic subjects can also rely on muscle MRI evaluation to detect early signs of the disease (Faraguna et al., 2023). On the other hand, the concept that biallelic IVS1 is not a severe genotype cannot restrict the access to available therapy for carriers presenting symptoms. Indeed, 80% of adult patients presenting the homozygous IVS1 genotype developed a mild to moderate progressive LOPD and should be considered for ERT treatment.

Beside ERT, dedicated therapeutic avenues are currently explored for this specific molecular defect. Indeed, different authors have achieved a partial correction of the IVS1-induced aberrant splicing by targeting nearby IVS1 genomic sequences with antisense oligonucleotides (Wal et al., 2017a; Wal et al., 2017b; Goina et al., 2017).

In our case, the identification of the IVS1 variant was fortuitous. Incidental parental findings during prenatal and reproductive genetic screening and testing have implications for reproductive decision making but also, as in this case, for parental health guidance and management. This example stresses the importance of pretest consultation and the relevance of clinical and ethical implications during prenatal genetic testing for the whole family group.

## Data Availability

The raw data supporting the conclusions of this article will be made available by the authors, without undue reservation.
