# Supplementary material for: Case Report: Incidental late-onset Pompe disease diagnosis in a man with no clinical and instrumental evidence of neuromuscular dysfunction
Source: Front Genet. 2025 Jun 23;16:1574381. doi: 10.3389/fgene.2025.1574381 (PMC12230033; doi:10.3389/fgene.2025.1574381)
Supplement: Supplementary file 1 [file Supplementaryfile1.pdf]

Supplementary Table 1 Clinical, biochemical and molecular findings in previously described subjects harboring the IVS1-32-13T>G *GAA* variant in homozygosis.

NA: not assessed; NBS: newborn screening; DBS: dried blood spot; y: years; m: months; I: increased; N: normal; +/+: homozygous; +/-: heterozygous.

Reference and Patient [Gender]	c.510C>T modifier	Creatine Kinase (U/L)	Clinical / Instrumental Findings	Age at symptom onset	Age at last follow up	Residual enzymatic activity compared to controls / cutoff value (%)
(1) (Patient 3) [F]	NA	N	Mild weakness (iliopsoas). Reduced deep tendon reflexes. EMG: myopathic	39 y	41 y	Muscle: 40%
(2) (Patient 17); (3) (Patient 12) [M]	NA	NA	Moderate myopathy. Respiratory Insufficiency (49 y). EMG: myopathic.	47 y	54 y	Muscle: <25%
(4) (Patient 20) [M]	NA	NA	Severe proximal weakness.	20 y	25 y	NA
(5-6)	NA	NA		Asymptomatic	NA	NA
(5-6)	NA	NA	Limb girdle weakness with severe respiratory involvement.	38 y	NA	NA
(5-6)	NA	NA	Limb girdle weakness with severe respiratory involvement.	40 y	NA	NA
(7) (Patient 16) [M]	NA	NA	Mild muscle weakness. Mild respiratory involvement.	13 y	26 y	Lymphocytes: 10%
(8) (Patient 8) [F]	NA	I (912)	Lower limb muscle weakness, Respiratory distress.	38 y	47 y	NA
(9) (Patient 17)	+/+	NA	NA	59 y	67 y	Fibroblasts: <20%
(9) (Patient 23)	-/-	NA		Asymptomatic	2 y (NBS)	NA
(9) (Patient 24)	-/-	NA		Asymptomatic	1 y (NBS)	NA
(9) (Patient 25)	-/-	NA		Asymptomatic	38 y	NA
(9) Patient 26)	-/-	NA		Asymptomatic	37 y	NA
(10) (Patient 6) [F]	NA	I (1000)	Scoliosis. EMG: normal.	Asymptomatic	75 y	Muscle: absent
(9) (Patient 18); (11) (Patient II.1) [M]	+/+	I (771)	Proximal limb weakness. Respiratory involvement (dyspnea).	48 y	64 y	DBS: 39%; Myoblasts <50%; Muscle: <20%
(9) (Patient 19); (11) (Patient I.1 [F]	+/-	I (448)	Severe muscle weakness. Respiratory involvement.	12 y	53 y	DBS: 36%; Muscle <20%

(9) (Patient 20); (11) (Patient V.1); (12) (Patient 29) [M]	-/-	I (838)	Myalgia and cramps. EMG: normal.	49 y	54 y	DBS: 5%; Fibroblasts: <20%
(9) (Patient 21); (11) (Patient III.1) [M]	-/-	I (540)	Proximal limb weakness. Myalgia.	58 y	62 y	DBS: 58%; Muscle: 12%
(9) (Patient 22); (11) (Patient IV.1) [F]	-/-	I (1663)	Proximal weakness.	42 y	46 y	DBS: 67%; Muscle <20%
(11) (Patient V.2); (12) (Patient 30) [M]	-/-	I (630)	EMG: normal.	Asymptomatic	42 y	DBS: 11%; Muscle: 13%
(13) (Patient 8)	NA	I (460-772)	Mild muscle weakness. Dyspnea.	48 y	60 y	NA
(13) (Patient 19)	NA	I (389)		Asymptomatic	14 y	NA
(14) (Patient 1) [F]	NA	N		Asymptomatic	34 y	NA
(14) (Patient 2) [F]	NA	N		Asymptomatic	1 y (NBS)	Lymphocytes: N (low level)
(15) (Patient 4) [F]	NA	I (366)	Subtle motor signs. Essentially asymptomatic.	4 m	13 m (NBS)	NA
(15) (Patient 5) [F]	NA	N	Subtle motor signs. Feeding difficulties. Essentially asymptomatic.	3 m	5 m (NBS)	NA
(15) (Patient 6) [M]	NA	N	Subtle motor signs. Essentially asymptomatic.	6 m	8 m (NBS)	NA
(15) (Patient 7) [F]	NA	N	Subtle motor signs. Essentially asymptomatic.	6 m	8 m (NBS)	NA
(16) (LOPD - Patient 1)	NA	I (530)		Asymptomatic	3 m (NBS)	DBS: 63%
(16) (LOPD - Patient 3)	NA	N		Asymptomatic	3 m (NBS)	DBS: 67%
(16) (LOPD - Patient 4)	NA	I (361)		Asymptomatic	3 m (NBS)	DBS: 45%
(16) (LOPD - Patient 5)	NA	I (333)		Asymptomatic	3 m (NBS)	DBS: 66%
(16) (LOPD - Patient 6)	NA	N		Asymptomatic	3 m (NBS)	DBS: 53%
(16) (LOPD - Patient 13)	NA	N		Asymptomatic	3 m (NBS)	DBS: 28%
(16) (LOPD - Patient 16)	NA	N		Asymptomatic	3 m (NBS)	DBS: 63%
(16) (LOPD - Patient 17)	NA	N		Asymptomatic	3 m (NBS)	DBS: 43%

(16) (LOPD - Patient 20)	NA	N		Asymptomatic	3 m (NBS)	DBS: 39%
(16) (LOPD - Patient 21)	NA	N		Asymptomatic	3 m (NBS)	DBS: 62%
(16) (LOPD - Patient 22)	NA	N		Asymptomatic	3 m (NBS)	DBS: 47%
(16) (LOPD - Patient 30)	NA	NA		Asymptomatic	3 m (NBS)	DBS: 51%
(17) (Patient 1) [F]	NA	NA	LOPD (not specified)	30 y	30 y	DBS: 70%
(17) (Patient 2) [M]	NA	NA	LOPD (not specified)	20 y	20 y	DBS: 65%
(17) (Patient 3) [F]	NA	NA	LOPD (not specified)	47 y	47 y	DBS: 33%
(17) (Patient 4) [F]	NA	NA	LOPD (not specified)	64 y	64 y	DBS: 38%
(17) (Patient 5) [M]	NA	NA	LOPD (not specified)	41 y	41 y	DBS: 35%
(18) (Patient 6)	NA	NA		Asymptomatic	NA (NBS)	NA
(18) (Patient 4) [F]	NA	NA		Asymptomatic	10 m (NBS)	NA
(18) (Patient 9)	NA	NA		Asymptomatic	NA (NBS)	NA
(18) (Patient 18)	NA	NA		Asymptomatic	NA (NBS)	NA
Our proband (II-1) [M]	-/-	N	EMG: normal.	Asymptomatic	33 y	Leukocytes: 7%

References

1. Sharma MC, Schultze C, von Moers A, Stoltenburg-Didinger G, Shin YS, Podskarbi T, et al. Delayed or late-onset type II glycogenosis with globular inclusions. Acta Neuropathol (Berl). 2005 Aug;110(2):151–7.

2. Schoser BGH, Müller-Höcker J, Horvath R, Gempel K, Pongratz D, Lochmüller H, et al. Adult-onset glycogen storage disease type 2: clinico-pathological phenotype revisited. Neuropathol Appl Neurobiol [Internet]. 2007 [cited 2024 Nov 6];33(5):544–59. Available from: https://onlinelibrary.wiley.com/doi/abs/10.1111/j.1365-2990.2007.00839.x

3. Müller-Felber W, Horvath R, Gempel K, Podskarbi T, Shin Y, Pongratz D, et al. Late onset Pompe disease: clinical and neurophysiological spectrum of 38 patients including long-term follow-up in 18 patients. Neuromuscul Disord NMD. 2007 Oct;17(9–10):698–706.

4. Nascimbeni AC, Fanin M, Tasca E, Angelini C. Molecular pathology and enzyme processing in various phenotypes of acid maltase deficiency. Neurology [Internet]. 2008 Feb 19 [cited 2025 Jan 12];70(8):617–26. Available from: https://www.neurology.org/doi/full/10.1212/01.wnl.0000299892.81127.8e

5. Semplicini C, Letard P, De Antonio M, Taouagh N, Perniconi B, Bouhour F, et al. Late-onset Pompe disease in France: molecular features and epidemiology from a nationwide study. J Inherit Metab Dis. 2018 Nov;41(6):937–46.

6. Laforêt P, Laloui K, Granger B, Hamroun D, Taouagh N, Hogrel JY, et al. The French Pompe registry. Baseline characteristics of a cohort of 126 patients with adult Pompe disease. Rev Neurol (Paris). 2013;169(8–9):595–602.

7. Herzog A, Hartung R, Reuser AJJ, Hermanns P, Runz H, Karabul N, et al. A cross-sectional single-centre study on the spectrum of Pompe disease, German patients: molecular analysis of the GAA gene, manifestation and genotype-phenotype correlations. Orphanet J Rare Dis [Internet]. 2012 Jun 7 [cited 2025 Jan 12];7(1):35. Available from: https://doi.org/10.1186/1750-1172-7-35

8. Niño MY, Mateus HE, Fonseca DJ, Kroos MA, Ospina SY, Mejía JF, et al. Identification and Functional Characterization of GAA Mutations in Colombian Patients Affected by Pompe Disease. JIMD Rep. 2013;7:39–48.

9. Bergsma AJ, In 't Groen SLM, van den Dorpel JJA, van den Hout HJMP, van der Beek NAME, Schoser B, et al. A genetic modifier of symptom onset in Pompe disease. EBioMedicine. 2019 May;43:553–61.

10. Echaniz-Laguna A, Carlier RY, Laloui K, Carlier P, Salort-Campana E, Pouget J, et al. SHOULD patients with asymptomatic pompe disease be treated? A nationwide study in france. Muscle Nerve [Internet]. 2015 [cited 2024 Nov 6];51(6):884–9. Available from: https://onlinelibrary.wiley.com/doi/abs/10.1002/mus.24653

11. Musumeci O, Thieme A, Claeys KG, Wenninger S, Kley RA, Kuhn M, et al. Homozygosity for the common GAA gene splice site mutation c.-32-13T>G in Pompe disease is associated with the classical adult phenotypical spectrum. Neuromuscul Disord [Internet]. 2015 Sep 1 [cited 2024 Nov 6];25(9):719–24. Available from: https://www.sciencedirect.com/science/article/pii/S0960896615006604

12. Montagnese F, Barca E, Musumeci O, Mondello S, Migliorato A, Ciranni A, et al. Clinical and molecular aspects of 30 patients with late-onset Pompe disease (LOPD): unusual features and response to treatment. J Neurol. 2015;262(4):968–78.

13. Vill K, Schessl J, Teusch V, Schroeder S, Blaschek A, Schoser B, et al. Muscle ultrasound in classic infantile and adult Pompe disease: a useful screening tool in adults but not in infants. Neuromuscul Disord NMD. 2015 Feb;25(2):120–6.

14. Golden-Grant K, Merritt II J l., Scott C r. Ethical considerations of population screening for late-onset genetic disease. Clin Genet [Internet]. 2015 [cited 2025 Jan 12];88(6):589–92. Available from: https://onlinelibrary.wiley.com/doi/abs/10.1111/cge.12566

15. Rairikar MV, Case LE, Bailey LA, Kazi ZB, Desai AK, Berrier KL, et al. Insight into the phenotype of infants with Pompe disease identified by newborn screening with the common c.-32-13T>G “late-onset” GAA variant. Mol Genet Metab [Internet]. 2017 Sep 19 [cited 2024 Nov 6];122(3):99. Available from: https://pmc.ncbi.nlm.nih.gov/articles/PMC5722675/

16. Ficicioglu C, Ahrens-Nicklas RC, Barch J, Cuddapah SR, DiBoscio BS, DiPerna JC, et al. Newborn Screening for Pompe Disease: Pennsylvania Experience. Int J Neonatal Screen. 2020 Nov 13;6(4):89.

17. Gal A, Grosz Z, Borsos B, Szatmari I, Sebők A, Jávor L, et al. Correlation of GAA Genotype and Acid-α-Glucosidase Enzyme Activity in Hungarian Patients with Pompe Disease. Life [Internet]. 2021 May 31 [cited 2024 Nov 6];11(6):507. Available from: https://pmc.ncbi.nlm.nih.gov/articles/PMC8228169/

18. Huggins E, Holland M, Case LE, Blount J, Landstrom AP, Jones HN, et al. Early clinical phenotype of late onset Pompe disease: Lessons learned from newborn screening. Mol Genet Metab [Internet]. 2022 Mar 1 [cited 2025 Jan 12];135(3):179–85. Available from: https://www.sciencedirect.com/science/article/pii/S1096719222000178
